# Suicidal Communication Prior to Suicide in Children and Young Adults—A Medical Records Review in Health Care Services in Sweden

**DOI:** 10.3390/ijerph22010031

**Published:** 2024-12-29

**Authors:** Anna-Lena Hansson, Per Johnsson, Sophia Eberhard, Anna Ehnvall, Sara Lindström, Margda Waern, Åsa Westrin

**Affiliations:** 1 Department of Child and Adolescent Psychiatry, Skåne County, SE-22185 Lund, Sweden; 2 Department of Psychology, Lund University, SE-22362 Lund, Sweden; 3 Department of Clinical Sciences, Child and Adolescent Psychiatry, Lund University, SE-22100 Lund, Sweden; 4 Department of Psychiatry and Neurochemistry, Institute of Neuroscience and Physiology, University of Gothenburg, SE-41345 Gothenburg, Sweden; 5 Psychiatric Outpatient Clinic, Region Halland, SE-43243 Varberg, Sweden; 6 Department of Clinical Sciences, Faculty of Medicine, Lund University, SE-22184 Lund, Sweden; 7 Office of Psychiatry and Habilitation, Region Skåne, SE-22363 Lund, Sweden; 8 Department of Psychotic Disorders, Sahlgrenska University Hospital, Region Västra Götaland, SE-41345 Gothenburg, Sweden; 9 Department of Clinical Sciences, Psychiatry, Lund University, SE-22184 Lund, Sweden; 10 The Region Skåne Committee on Psychiatry, Habilitation and Technical Aids, SE-20501 Malmö, Sweden

**Keywords:** suicidal communication, suicidal ideation, suicide, children, young adults, psychology, clinical implications, public health, Sweden

## Abstract

Suicide among children and young adults is a leading cause of mortality, highlighting the importance of the development of life-saving interventions. This study is part of the nationwide study Retrospective investigation of health care utilization of individuals who died by suicide in Sweden in 2015, Lund University, Sweden. The aim was to gain a better understanding of verbal suicidal communication and suicidal behaviour in children and young adults who die by suicide, to analyse gender and age differences, and to discuss the findings in relation to the prevailing psychological theories of suicidality. The study sample consisted of medical records from final health care consultations of 114 individuals up to 25 years, who died by suicide in Sweden a single year. Suicidal plans were documented in 13 percent of children and young adults. Females were more likely to have a notation of suicidal communication than males. Twenty-seven percent had made previous suicide attempts. Approximately 90 percent of the study cohort had contact with health care settings within 24 months prior to suicide. Questioning about suicidal plans appears to be an insufficient tool to assess suicidality in children and young adults. Clinical implications regarding alternative assessment methods and preventive measures are discussed.

## 1. Introduction

Suicide among children and young adults constitutes a global health concern, ranked as one of the five leading causes of mortality in the 10–24 age group in the Global Burden of Disease Regions, except for sub-Saharan Africa [1]. In Sweden, the prevalence of suicide is 1.1 in 100,000 in children and adolescents up to 18 years, with 96 percent of cases occurring among adolescents aged 13–17 years [2]. Suicide accounts for approximately 10 percent of deaths in children and young adults (0–24 years) in Sweden [3]. The communication of suicidal thoughts and behaviour is a cornerstone of suicide prevention as it represents the first step in accessing help [4]. However, the degree to which suicidal ideation and behaviours are communicated to care professionals prior to suicide needs to be clarified.

Several previous studies address the rate of help-seeking the year before suicide [5,6,7,8]. Based on a review of ten studies, about 71 percent of individuals under 25 had contact with primary health care in the year leading up to death by suicide [6]; the kind of primary health care or reason for contact were not specified. In one study, 88 percent of children and young adults had a health care visit in the year prior to death by suicide, with the findings of all the health care subtypes being more frequent among the suicide decedents than among their counterparts in the control group [8], underscoring the opportunity of providing efficient suicide prevention within the clinic.

A communication of suicidal ideation is often seen as a gateway to the assessment of whether a person is suicidal [9]. However, reports of suicidal ideation are shown to have a very low predictive utility in relation to deaths by suicide [10]. Suicide risk assessment based on suicidal communication of the level of planning has no empirical support; the reliability and validity of suicide risk formulation are dependent on the robustness of the suicide risk assessment [10]. Patients may deny suicidal ideation for several reasons. Suicidal thoughts come in many forms; hence some phrasings may disguise other meanings. Furthermore, the self-reporting of serious suicidal communication of attempting suicide has little correlation to the communication of a history of suicide attempts within the last year, and also a low correlation to whether a youth will die by suicide [10]. The overwhelming focus on suicide risk assessment measures is problematic, we need to abandon this endeavour of risk prediction [11]. An alternative approach is to invest time in gaining therapeutic alliance rather than ticking boxes, and uncover modifiable risk-factors and unmet needs, in an individualized way [12].

In an interview study [13] with suicide attempt survivors regarding verbal suicidal communication prior to suicide attempt, some expressed that they had felt unable to share their inner world, and others chose purposely not to share. Suicidality and self-destructive thoughts may also be expressed through action [14]. In a study of 155,516 people aged 10 years and older who died by suicide between 2013 and 2019, only about five percent of those who disclosed suicidal thoughts and behaviours the last month had communicated that to a health care professional the month prior to suicide [4]. Although many individuals seek help prior to suicide, studies on communication of suicidality to a health care professional are rare, specifically regarding children and young adults and their communication of suicidality prior to suicide. Since previous studies show low predictive utility in the effectiveness of preventing suicide based on suicidal communication, there is a need to find alternative ways to prevent suicide. Very few previous studies about suicidal thoughts and behaviours with samples including adolescents have included suicide deaths as an outcome [15]. Franklin et al. [15] pinpoint the need for further studies to make advances in the understanding of suicidal thoughts and behaviours and to make advances in the clinical relevance of the findings.

The focus of this study is to describe the frequencies of the communication of suicidality prior to suicide in a national cohort in Sweden and to apply the theoretical psychological approaches to the issue of reducing suicidality in children and young adults to our findings.

### Aim

The aim of this study was to gain a better understanding of suicidal communication and behaviour prior to suicide in children and young adults as documented in medical records, specifically (1) to measure suicidal communication and behaviour leading up to suicide in children and young adults (2) to examine age and gender differences in suicide, suicidal communication, and suicidal behaviour, and (3) to apply psychological theories about suicidality in children and young adults to the findings.

## 2. Materials and Method

This study is part of the nationwide project Retrospective Investigation of health care Utilization of individuals who died by suicide in Sweden in 2015, Lund University, Sweden [16]. The data are stored at Forum South, Clinical Studies, Sweden in collaboration with Lund University, Sweden. This is a medical records review that covers all individuals who died by suicide in Sweden a single year.

### 2.1. Sample

In this sub-study, the study cohort consist of children and young adults up to 25. In 2015, 114 individuals aged 24 and younger were registered as deaths by suicide [3]. These 24 children (0–17 years) and 90 young adults (18–24 years) constitute the cohort of the current study. Medical records from psychiatric services, primary health care, and specialized somatic health care were examined. The catchment area for the current sub-study also includes the Stockholm region, which was not included in the previous studies in this project [16,17].

### 2.2. Data Collection Process

Data were retrieved from all available medical records for each individual record 24 months prior to death by suicide. Data were available from all public records and efforts were made to obtain records from private clinics. The portion of private clinics varied between regions; a survey was sent out to the record reviewers, and they responded that the problems of accessing non-governmental health care records were minimal. A structured investigation protocol was constructed based on guidelines from the Swedish Psychiatric Organisation [18]. In each of the 21 Swedish counties, one to three professionals were engaged as record reviewers and trained with the investigation protocol. Every county selected their record reviewers; in total, there were 49 record reviewers. The research group provided a manual with frequently asked questions and were available to discuss further questions. Medical records were reviewed during 2016–2020 for each person who died by suicide, including a broad range of variables [16,17,19]. Variables pertaining to suicidal communication are in focus in this sub-study. Included were notes at the last visit to any health care professional, the last visit to a physician, and the last visit to in-patient care in notes by health care staff. These notes were included from each health care unit the suicide descendant visited within 24 months prior to suicide.

### 2.3. Variables

Variables of death wishes, suicidal thoughts, and suicidal plans noted in the medical records of psychiatric services, primary care, and specialized somatic health care were used as a measure of suicidal communication. Death wishes, suicidal thoughts, and suicidal plans were coded by the reviewers if they were noted in the records, using the ordinary terms in the Swedish medical settings referring to death wishes, suicidal thoughts, and suicidal plans. Death wishes meaning wanting to die, suicidal thoughts meaning thinking about committing suicide, and suicidal plans meaning having plans about committing suicide. Variables regarding noted death wishes, suicidal thoughts, and suicidal plans from these different public authorities were grouped into three variables named death wish, suicidal thoughts, and suicidal plans, respectively.

Variables about previous suicide attempt noted in any health care setting and previous non-suicidal self-injury noted in any health care setting were used as measures of suicidal behaviours prior to suicide.

Variables about visits to health care in the last 24 months were used to give a background of how many of the 114 individuals visited psychiatric services, primary care, and specialized somatic health care during the 24 months prior to suicide, including the number of days from the final visit to any health care setting. Variables of visits to health care were used as a measure of help-seeking; these final visits within 24 months prior to death by suicide were included, regardless of reason for contact and regardless of disclosure of suicidality.

Demographic variables of age and gender were used. Age was based on birthday and time of death. The individuals were divided into children (0–18 years) and young adults (18–24 years). Variables of gender were based on the Swedish personal identity number at the time of death, indicating male or female. None of the individuals in this cohort had a registered gender change.

### 2.4. Data Analysis

Descriptive statistics were calculated for all variables (frequencies and percentages). Chi-square test was used for significance at 95 percent level between males and females, and between the age groups of children and young adults. Significant differences in the frequency of the variables between the groups were calculated. Chi-square was chosen as the best option of statistics, due to low numbers of individuals and observations. Small numbers were excluded in accordance with general data protection regulation in EU (GDPR). IBM SPSS Statistics version 28.0.1.0 (142) was used for the analysis.

### 2.5. Ethical Considerations

According to the Swedish Act Concerning the Ethical Review of Research Involving Humans (203:460) and an advisory statement from the Regional Ethical Review Board in Lund, Sweden (Ethical Review Decision no. 2017/234), ethical review is not relevant in the *Retrospective investigation of health care utilization of individuals who died by suicide in Sweden 2015* because as it does not include living human participants. The inclusion of data from Stockholm County was approved by the Swedish Ethical Review Authority (Ethical Review Decision no. 2019-02792).

## 3. Results

### 3.1. Age and Gender Distribution

A total of 114 individuals aged 24 and under died by suicide in Sweden in 2015, 71 males and 43 females, age range 11–24. There were 24 children (range 11–17 years) and 90 young adults (age range 18–24 years). The 24 children included 13 boys and 11 girls. The 90 young adults included 58 young men and 32 young women (Table 1).

### 3.2. Verbal Suicidal Communication and Visits to Health Care by Gender

The variables regarding suicidal communication; death wishes, suicidal thoughts, and suicidal plans were analysed. Any form of verbal suicidal communication was noted in a third of the total cohort, 23 percent of males and 47 percent of females. Death wishes were noted in almost a fifth of the total cohort, 15 percent of males and 26 percent of females. Suicidal thoughts were noted in almost a third of the total, 21 percent of males and 42 percent of females. Suicidal plans were noted in 13 percent of the study population, 7 percent of males and 23 percent of females. There were significant differences between males and females in the frequencies of suicidal communication of suicidal thoughts, suicidal plans any in any suicidal communication; females had significantly more suicidal communication noted. Any suicidal communication or previous suicide attempt were noted in total in 42 percent, 31 percent of males and 60 percent of females. Previous suicide attempts were noted in total in 27 percent, 20 percent in males and 40 percent in females. Previous non-suicidal self-injury was noted in total in 28 percent, 21 percent of males and 40 percent of females. Any suicidal communication and behaviours including non-suicidal self-injury was noted in approximately half of the cohort, 37 percent of males and 70 percent of females. The differences were significant between males and females regarding suicidal behaviours. Notes of both previous suicide attempts *and* any verbal suicidal communication were present in 18 percent of total cases, 13 percent of males and 26 percent of females.

Visits to one or more health care setting (psychiatric care, primary care, or specialized somatic health care) in the last two years were noted in total in 89 percent; 86 percent of males and 95 percent of females. Over half of study cohort had a notation of contact with psychiatric services; 54 percent of males and 70 percent of the females. Contact with primary care was noted in total in total in 69 percent; 63 percent of males and 79 percent of females. Specialized somatic health care visits were noted in over half of the cohort; 54 percent of males and 58 percent of females. The final visit per individual 24 months prior to suicide took place in psychiatric services in 39 percent (35% in males and 47% in females), 30 percent in primary care (28% in males and 33% in females), and 22 percent in specialized somatic health care (24% in males and 19% in females). Significantly more females than males had a last visit within the last month prior to suicide. The final visit took place within the last month for 56 percent of all the suicide decedents (46% of males, 72% of females), within the last year for 82 percent (73% of males and 95% of females), and within the last two years for 89 percent (86% of males and 95% of females). See Table 2 for the gender groups and the total number of individuals 0–24 years.

### 3.3. Verbal Suicidal Communication and Visits to Health Care by Age Group

Divided into age groups, 21 percent of children and 31 percent of young adults had suicidal thoughts noted. The numbers regarding death wishes and suicidal plans are not disclosed in the age groups, due to small numbers in the group of children. Previous suicide attempts were noted in total in 27 percent; numbers by age groups are not disclosed due to small numbers in children. Previous non-suicidal self-injury were noted in 25 percent of children and 29 percent of young adults. Combined into any suicidal communication and behaviours including non-suicidal self-injury, 42 percent of children and 51 percent of young adults had notes of at least one of those. With non-suicidal self-injury excluded, 29 percent of children and 46 percent of young adults had any form of suicidal communication or previous suicide attempt noted. Notes of both previous suicide attempt or non-suicidal self-injury and any form of suicidal communication were noted in a fifth of the study cohort.

A total of 92 percent of children and 89 percent of young adults had contact with one or more of psychiatric services, primary health care, or specialized somatic health care 24 months prior to death by suicide. A total of 63 percent of children and 59 percent of young adults had contact with psychiatric services. Contact with primary care was noted in 58 percent of children and 72 percent of young adults. Specialized somatic health care consultations were noted in 50 percent of children and 57 percent of young adults. The final visit was in the psychiatric services for 46 percent of the children and for 38 percent of the young adults; in primary care for 25 percent of the children and for 31 percent of the young adults; and in specialized somatic health care for 25 percent of children and 21 percent of the young adults. The final visit took place the last month for 50 percent of the children and 58 percent of young adults; the last year for 83 percent of the males and 81 percent of the females; and the last two years for 92 percent of the children and 89 percent of the young adults. See Table 3 for results by age groups and the total number of individuals 0–24 years.

## 4. Discussion

Approximately 90 percent of children and young adults had contact with psychiatric services, primary health care, or specialized somatic care in the 24 months prior to their death. However, only a minority explicitly communicated their suicidality to a health care professional, with about 30 percent having any form of suicidal communication noted, and only 13 percent specifically communicating suicidal plans, as documented in their medical records. The help-seeking rates in this study are similar to those found in a previous Swedish study that included individuals of all ages, where 86 percent had contact with health care services within a year before suicide, compared to 69 percent in the general population during the same period [7].

In this current study, notes of death wishes were present in 15 percent of males and 26 percent of females, and suicidal thoughts were present in 21 percent of males and 42 percent of females. Suicidal thoughts are common in the general population [10], which may lead to their severity being underestimated or misinterpreted.

In the current study, males and females differed in both suicidal thoughts and behaviours, as well as help-seeking in the month prior to suicide. Females were more likely to have visited health care in the month prior to suicide and were more likely to have reported suicidal thoughts and behaviours in their last health care visits within two years of suicide. This could have implications for how health care providers approach the topic of suicide with females versus males. The gender and age distribution in the study cohort is representative for the suicide deaths in the 0–24 years-age group over the last 25 years [3]. The population of child suicide decedents of 2015 in this study shows no significant gender differences in the prevalence among children and young adults; this aligns with the Swedish study of death by suicide in children (0–17 years) between 2000 and 2018 [2], which also showed almost no gender differences in children regarding prevalence.

This Swedish study shows rates of differentiated measures of suicidal communication and behaviours, with one-third of the individuals who died by suicide having suicidal thoughts or any form of verbal suicidal communication noted. When focusing specifically on suicidal plans noted, considered to be the most severe form of verbal suicidal communication, the numbers are in our study cohort 13 percent; in 7 percent of males and in 23 percent of females.

To come as close as possible with our data to the concept of suicidal thoughts and behaviours used in the literature, we can combine our measures of any form of suicidal communication and previous suicide attempt; any of these were noted in total in 42 percent of this study population; in the sub-groups in 31 percent of males, in 60 percent of females, in 29 percent of children, and in 46 percent of young adults, during the lasts visits 24 months prior to suicide. Over half of the cohort had a last visit within the last month (46% of males and 72% of females).

Though not fully comparable to the study by Clark and Blosnich et al. [4], due to different measures and different health care settings, the numbers from our study are higher in the combined form of any suicidal communication or behaviour. If comparing our measure of noted suicidal plans, the numbers are closer. They reported on to whom suicidal thoughts and behaviours the last month prior to suicide were disclosed. Their result was that approximately five percent of all ages disclosed suicidal thoughts and behaviours to a health care professional of the quarter who had disclosed suicidal thoughts and behaviours to anyone the month prior to suicide [4].

As Silverman and Berman have shown, there is no empirical evidence that active suicidal expression indicates a higher risk than passive expression. The conclusion from our findings might be that it is important to mind both death wishes and suicidal thoughts, regardless of a disclosed plan. Given the reporting rate of any form of suicidal communication, priority should be placed on improving health care to reduce missed opportunities for intervention. However, the low reporting rate of suicidal plans in this study suggests that relying on the communication of such plans is an insufficient tool for assessing severe suicidality among children and young adults.

The medical history of the children and young adults who died by suicide in our study demonstrate that individuals at serious risk require more extended help to be understood, beyond voluntary disclosures or direct questioning about suicidality. This could be provided for in the following ways: Preventive interventions within health care services need to proactively reach those in need [20]. Chitty et al. (2023) argue that there is a need for a focus shift, from heavily relying on at-risk individuals’ help-seeking to suicide prevention intervention [20], that is, increased focus on preventive interventions. High-quality psychiatric health care for children and strengthening of family relations are required as means to prevent suicide [2]. Teens most in need of mental health care are reluctant to seek help, often due to stigma and negative beliefs toward mental health services and professionals [21].

Given the result of our study, as well as many other studies, help-seeking behaviour among children and young adults is relatively high in the year preceding suicide. This suggests that suicide prevention interventions can be effectively expanded both within health care services and beyond.

### 4.1. Clinical Implications

The clinical implications following our findings of high help-seeking prior to suicide in combination with low reporting rate of suicidal plans will be discussed in relation to the prevailing psychological theories of suicidality. Psychological theories of suicidality emphasize psychological aspects relevant to save lives. Suicide preventions are needed at school ages and post-school ages, gender-specific prevention and effective preventive strategies are needed, and these interventions need to follow a personalized approach [22]. To increase the likelihood of young patients sharing their suicidal plans, it is important to be aware that an atmosphere of misrecognition in treatment can lead to suicidal protest and escape [23,24]. Russel et al. underline that it is critical to address the known risk factors for suicide with interventions [25]. The psychological key concepts of suicidality in youth have the possibility to guide towards understanding and problem-solving in meeting suicidal children and young adults, with the goal to save young people’s lives: perceived burdensomeness and thwarted belongingness [26], psych ache [27], the fluctuating nature of suicidality [28], psychological development of the self [29,30], the role of parental styles [31,32,33,34], and subconscious communication in self-destructive behaviour [14].

There is a need to look for alternative ways of preventing suicide. To diminish the missed opportunities for suicide prevention, better assessments and interventions are needed [5,35,36]. The children and young adults who died by suicide had to a high extent (89% during the last 24 months) reached out to the health care system, but 71 percent of them did not have thoughts about contemplating suicide noted and 87 percent did not have suicidal plans noted, which underscores that alertness to what is not communicated is needed. Non-verbal self-destructive behaviour and emotional or physical withdrawal can imply suicidal ideation conveyed non-verbally through actions [14]. The non-verbal communication is important to pay attention to.

In the perspective of trying to understand the low rate of noted suicidal plans in the suicide decedents’ records in our findings, understanding ambivalence is an important aspect. Patients are often ambiguous: It is possible to want to die and not want to die, to want help and not want help at the same time [27]. To manage this ambiguity, we can use psychological theory and put it into practical use in health care settings. Children and young adults may be ambivalent when asked about suicide plans. A study about the fluctuating nature of suicidality [28] shows that suicidality fluctuates over shorter and longer periods, even over hours. Another plausible explanation for why they did not tell, regardless if they were asked or not, are barriers such as stigma and negative beliefs towards mental health services and professionals, which was the case in young people not seeking help for mental health problems in a previous study [21]. Those who did disclose having suicidal plans might have had previous positive experiences with services. A patient talking about suicidal plans, suicidal thoughts, and death wishes is considered a risk factor, but not telling does not necessarily indicate an absence of suicidal thoughts and plans. In the study of Clark and Blosnich [4], the numbers of disclosure of suicidal thoughts and behaviour prior to suicide of those who had told someone were higher to a previous or current partner, to a parent, or to a friend or colleague. Between 10–50 percent had told someone in a private relationship, as opposed to around five percent telling a health care professional [4]. We can learn from that and take information of suicidality from relatives seriously, even though the patient is not able or not willing to share.

Psychological theories about suicidality argue that suicidal thoughts to a high extent are preceded by strain and emotional load [26,27,31,32,33,34]. There are several reasons why questions about death wishes, suicidal thoughts, and suicidal plans might be hard to answer, and it might even be the case that they are hard to ask. Maybe it would be easier to talk about ongoing strain and ongoing emotional load; in those cases, the outspoken communication of suicidality is not a passable road. We need to be aware of suicidality based on other input than verbal suicidal communication; for example, a life situation with conflicts, emptiness, loneliness, and hopelessness that are hard to handle (see [31]). Russel et al. [25] underlines that it is important to address the risk factors for suicide with interventions. To focus on how to help and how to comfort the suffering and struggling person and identifying unmet needs might be more important than the questioning and assessment of suicidal risk [5,35,36].

Reducing feelings of burdensomeness and improving feelings of belongingness are important factors according to the interpersonal theory of suicide [37,38,39]. The subconscious communication in self-destructive behaviour [14] is also relevant to take into account, as children and young adults may communicate suicidality and self-destructive thoughts through their actions, even if they do not report suicidality verbally. An atmosphere of misrecognition in treatment can lead to suicidal protest and escape, which is a factor to be aware of, and to be sure to do the opposite [23,24]. Forte et al. emphasize that the interventions need to follow a personalized approach [22], which is an important factor in improving the health care system. Regarding children, it is also important to mind dominating, abusive, and dismissive parental styles that correlate with suicide, and to intervene to prevent suicidality [31,32,33,34].

The medical records are used frequently to get an overview of the patient in daily care. Therefore, it is important that we have knowledge to understand that suicidal plans were not noted in the medical records of most of those children and young adults who died by suicide. Health care professionals may need to read between the lines and analyse what information might be lacking or being hard for the patient to tell and share.

The time has come to significantly change suicide risk assessment and suicide risk formulation. Instead, the treatment of the patient should be the foremost consideration in the clinicians’ mindset [9]. The clinical implications, when a majority of suicidal patients do not communicate suicidal plans, are to personalize the interventions, address the known risk factors, create a good atmosphere in the meetings, consider the subconscious communication, give hope, reduce the feeling of being a burden, improve the feeling of belongingness, and build trust in others and oneself in being able to cope with a troublesome situation including family relations.

### 4.2. Limitations

The sample of this study covers a national cohort of children and young adults who died by suicide in Sweden in a particular year, which gives high generalisability. Our study did not have a control group. Our sample included only those who died by suicide, which is a limitation to our study in not having the data of death wishes, suicidal thoughts, suicidal plans, and previous suicide attempts of those who did not die. The data from medical records have obvious limitations, with several possible reasons why some information is lacking, including insufficient queries to the patient, incomplete answers from the patient, and incomplete or inaccurate documentation in the medical record. There is a limitation of the data in not saying when within the timeframe of 24 months the verbal suicidal communication took place. Medical records from all private health care settings were not available; the amount of missing data due to private care records not included is unknown, though the reviewers valued the effect of missing data on the results presumably negligible. Only suicides due to determined intent were included, which means that some suicides were likely to have been missed. The results of our study cannot be extrapolated to settings with less access to health care for children and young adults.

### 4.3. Research Implications

Implications for future studies are to further explore the communication of children and young adults around suicidality, and to improve assessment and interventions to save lives. Our future studies will further investigate the help-seeking behaviour and suicidal communication of children and young adults prior to suicide and how suicidal ideation prior to suicide is expressed and met in the health care system and will include studying the meaning for the patient of communicating with a professional about suicidal ideation and suicidal behaviour. Future studies will incorporate various theoretical perspectives, to deepen the understanding of suicidality in children and young adults. Replication of our study is warranted.

## 5. Conclusions

This study is to our knowledge the first to systematically analyse suicidal communication in all children and young adults who died by suicide in Sweden during a single year. Findings are based on a unique dataset retrieved from each patient’s medical records, previously assembled by the research group. The study adds new and vital information of relevance for clinicians who meet, assess, and treat young suicidal patients. Only thirteen percent of the cohort aged 24 and under communicated suicidal plans, suggesting that other methods are needed to aid clinician assessment and management of suicidal patients. Further research is warranted, including replication of our findings in other settings. There is a growing need to reconsider and improve suicide risk assessment and formulation. The focus should shift from solely evaluating risk to prioritizing the patient’s treatment and care.

## Figures and Tables

**Table 1:** Number and proportions of children and young adults who died by suicide in Sweden 2015, male and female.

	Children *n* (%)	Young Adults *n* (%)
Male	13 (54%)	58 (64%)
Female	11 (46%)	32 (36%)
Total	24	90

Differences between children and young adults were tested with Chi-square tests and did not denote significance at a 5% level.

**Table 2:** Number and percentage (%) of individuals aged under 25 years with notation of suicidal communication, suicidal behaviour, and visits to psychiatric services, primary care, or specialized somatic health care 24 months prior to suicide, by gender (males *n* = 71, females *n* = 43).

	Males *n* (%)	Females *n* (%)	Total *n* (%)
Suicidal Communication			
- Death wish	11 (15%)	11 (26%)	22 (19%)
- Suicidal thoughts *	15 (21%)	18 (42%)	33 (29%)
- Suicidal plans *	5 (7%)	10 (23%)	15 (13%)
- Any of the above *	17 (23%)	21 (47%)	38 (33%)
Suicidal behaviours			
- Previous suicide attempt *	14 (20%)	17 (40%)	31 (27%)
- Previous non-suicidal self-injury (NSSI) *	15 (21%)	17 (40%)	32 (28%)
- Any of the above *	20 (28%)	24 (56%)	44 (39%)
Any Suicidal Communication and Behaviours			
- Including NSSI *	26 (37%)	30 (70%)	56 (49%)
- Excluding NSSI *	22 (31%)	26 (60%)	48 (42%)
Both of any Suicidal Communication and Behaviours			
- Including NSSI *	11 (15%)	14 (33%)	25 (22%)
- Excluding NSSI	9 (13%)	11 (26%)	20 (18%)
Health Care Setting last 24 Months			
- Contact with psychiatric services	38 (54%)	30 (70%)	68 (60%)
- Contact with primary care	45 (63%)	34 (79%)	79 (69%)
- Contact with specialized somatic health care	38 (54%)	25 (58%)	63 (55%)
- Any of the above ^2^	61 (86%)	41 (95%)	102 (89%)
Health Care Setting Final Visit before Suicide within 24 Months			
- Psychiatric services	25 (35%)	20 (47%)	45 (39%)
- Primary care	20 (28%)	14 (33%)	34 (30%)
- Specialized somatic health care	17 (24%)	8 (19%)	25 (22%)
- Any of the above ^2^	61 (86%)	41 (95%)	102 (89%)
Time of Final Visit to any Health Care Setting before Suicide			
Last month *	33 (46%)	31 (72%)	64 (56%)
Last year ^2^	52 (73%)	41 (95%)	93 (82%)
Last two years ^2^	61 (86%)	41 (95%)	102 (89%)

Differences between males and females were tested with Chi-square tests, * denotes significance at a 5% level. ^2^ Denotes that Chi-square tests were not done due to high/low numbers.

**Table 3:** Number and percentage (%) of children (0–17 years, *n* = 24) and young adults (18–24 years, *n* = 90) with a notation of suicidal communication, suicidal behaviour, and visits to psychiatric services, primary care, or specialized somatic health care 24 months prior to suicide, by age group.

	Children *n* (%)	Young Adults *n* (%)	Total *n* (%)
Suicidal Communication			
- Death wish ^1,2^	-	-	22 (19%)
- Suicidal thoughts	5 (21%)	28 (31%)	33 (29%)
- Suicidal plans ^1,2^	-	-	15 (13%)
- Any of the above	6 (25%)	32 (36%)	38 (33%)
Suicidal Behaviour			
- Previous suicide attempt ^1,2^	-	-	31 (27%)
- Previous non-suicidal self- injury (NSSI)	6 (25%)	26 (29%)	32 (28%)
- Any of the above	6 (25%)	38 (42%)	44 (39%)
Any of Suicidal Communication and Behaviour			
- Including NSSI	10 (42%)	46 (51%)	56 (49%)
- Excluding NSSI	7 (29%)	41 (46%)	48 (42%)
Both of any Suicidal Communication and Suicidal Behaviour			
- Including NSSI ^1,2^	-	-	25 (22%)
- Excluding NSSI ^2^	0 (0%)	20 (22%)	20 (18%)
Health Care Setting			
- Contact with psychiatric services	15 (63%)	53 (59%)	68 (60%)
- Contact with primary care	14 (58%)	65 (72%)	79 (69%)
- Contact with specialized somatic health care	12 (50%)	51 (57%)	63 (55%)
- Any of the above ^2^	22 (92%)	80 (89%)	102 (89%)
Health Care Setting Final Visit before Suicide within 24 Months			
- Psychiatric services	11 (46%)	34 (38%)	45 (39%)
- Primary care	6 (25%)	28 (31%)	34 (30%)
- Specialized somatic health care	6 (25%)	19 (21%)	25 (22%)
- Any of the above ^2^	22 (92%)	80 (89%)	102 (89%)
Time of Final Visit to any Health Care Setting before Suicide			
Last month	12 (50%)	52 (58%)	64 (56%)
Last year ^2^	20 (83%)	73 (81%)	93 (82%)
Last two years ^2^	22 (92%)	80 (89%)	102 (89%)

^1^ Some results are omitted due to small numbers in children, in accordance with GDPR (EU, 2016/679). Difference between children and young adults are tested with chi-square tests, no significant differences at a 5% level were found. ^2^ Denotes that chi-square tests were not done due to the high/low numbers.

## Data Availability

Data will be available on reasonable request.
